# Editorial Comment: Anticholinergic Drug Exposure and the Risk of Dementia: A Nested Case-Control

**DOI:** 10.1590/S1677-5538.IBJU.2020.02.10

**Published:** 2020-01-10

**Authors:** Marcio Augusto Averbeck

**Affiliations:** 1 Unidade de Videourodinâmica Hospital Moinhos de Vento Porto AlegreRS Brasil Chefe de Neuro-Urologia, Unidade de Videourodinâmica, Hospital Moinhos de Vento. Porto Alegre, RS, Brasil

Coupland CAC ^1^, Hill T ^1^, Dening T ^2^, Morriss R ^2^, Moore M ^3^, Hippisley-Cox J ^1,4^

^1^ Division of Primary Care, University of Nottingham, Nottingham, England; ^2^ Division of Psychiatry and Applied Psychology, Institute of Mental Health, Nottingham, England; ^3^ University of Southampton Medical School, Primary Care and Population Sciences, Aldermoor Health Centre, Southampton, England; ^4^ Nuffield Department of Primary Care Health Sciences, University of Oxford, Oxford, England

JAMA Intern Med. 2019 Jun 24. [Epub ahead of print]

**DOI:** 10.1001/jamainternmed.2019.0677 | **ACCESS:** 10.1001/jamainternmed.2019.0677

## COMMENT

There is increasing evidence for a possible causal association between the chronic use of anticholinergics and the risk of developing dementia. The inherent limitation for establishing such causal nexus is precisely the fact that the prevalence of overactive bladder (OAB) symptoms and the prescription of antimuscarinics tends to increase in older individuals, exactly in the same age groups in which the incidence of dementia is higher (1).

Coupland et al. carried out a nested case-control study based on the QResearch primary care database from England, including 58 769 patients with a diagnosis of dementia and 225 574 controls 55 years or older matched by age, sex, general practice, and calendar time. Primary exposure was defined as the total standardized daily doses (TSDDs) of anticholinergic drugs prescribed in the 1 to 11 years prior to the date of diagnosis of dementia. There were significant increases in dementia risk for the anticholinergic antidepressants (adjusted OR [AOR], 1.29; 95%CI, 1.24-1.34), antiparkinson drugs (AOR, 1.52; 95%CI, 1.16-2.00), antipsychotics (AOR, 1.70; 95% CI, 1.53-1.90), bladder antimuscarinic drugs (AOR, 1.65; 95%CI, 1.56-1.75), and antiepileptic drugs (AOR, 1.39; 95%CI, 1.22-1.57) all for more than 1095 TSDDs. Associations were stronger in cases diagnosed before the age of 80 years.

Despite attempts to control for confounding variables, the results of case-control studies should be viewed with caution. Even so, this is not the first study to suggest an association between the chronic use of anticholinergics and the development of dementia. Physicians should be aware of new evidences and attempt to reduce exposure to anticholinergic drugs in middle-aged and older people. One question still needs to be answered: are all antimuscarinics alike?
